# Molecular Genetic Research and Genetic Engineering of *Taraxacum kok-saghyz* L.E. Rodin

**DOI:** 10.3390/plants12081621

**Published:** 2023-04-12

**Authors:** Bulat Kuluev, Kairat Uteulin, Gabit Bari, Elvina Baimukhametova, Khalit Musin, Alexey Chemeris

**Affiliations:** 1 Institute of Biochemistry and Genetics of UFRC RAS, 71 Pr. Oktyabrya, 450054 Ufa, Russia; 2 Institute of Plant Biology and Biotechnology, St. Timiryazev 45, 050040 Almaty, Kazakhstan; 3 Laboratory of Microclonal Propagation of Plants, Kazakh National Agrarian Research University, St. Valikhanov 137, 050000 Almaty, Kazakhstan

**Keywords:** *Taraxacum kok-saghyz*, natural rubber, genome, transcriptome, proteome, *cis*-prenyltransferase, rubber elongation factor, small rubber particle protein, inulin, fructan:fructan-1-fructosyltransferase

## Abstract

Natural rubber (NR) remains an indispensable raw material with unique properties that is used in the manufacture of a large number of products and the global demand for it is growing every year. The only industrially important source of NR is the tropical tree *Hevea brasiliensis* (Willd. ex A.Juss.) Müll.Arg., thus alternative sources of rubber are required. For the temperate zone, the most suitable source of high quality rubber is the Russian (Kazakh) dandelion *Taraxacum kok-saghyz* L.E. Rodin (TKS). An obstacle to the widespread industrial cultivation of TKS is its high heterozygosity, poor growth energy, and low competitiveness in the field, as well as inbreeding depression. Rapid cultivation of TKS requires the use of modern technologies of marker-assisted and genomic selection, as well as approaches of genetic engineering and genome editing. This review is devoted to describing the progress in the field of molecular genetics, genomics, and genetic engineering of TKS. Sequencing and annotation of the entire TKS genome made it possible to identify a large number of SNPs, which were subsequently used in genotyping. To date, a total of 90 functional genes have been identified that control the rubber synthesis pathway in TKS. The most important of these proteins are part of the rubber transferase complex and are encoded by eight genes for *cis*-prenyltransferases (*TkCPT*), two genes for *cis*-prenyltransferase-like proteins (*TkCPTL*), one gene for rubber elongation factor (*TkREF*), and nine genes for small rubber particle proteins (*TkSRPP*). In TKS, genes for enzymes of inulin metabolism have also been identified and genome-wide studies of other gene families are also underway. Comparative transcriptomic and proteomic studies of TKS lines with different accumulations of NR are also being carried out, which help to identify genes and proteins involved in the synthesis, regulation, and accumulation of this natural polymer. A number of authors already use the knowledge gained in the genetic engineering of TKS and the main goal of these works is the rapid transformation of the TKS into an economically viable rubber crop. There are no great successes in this area so far, therefore work on genetic transformation and genome editing of TKS should be continued, considering the recent results of genome-wide studies.

## 1. Introduction

**Figure 1:**
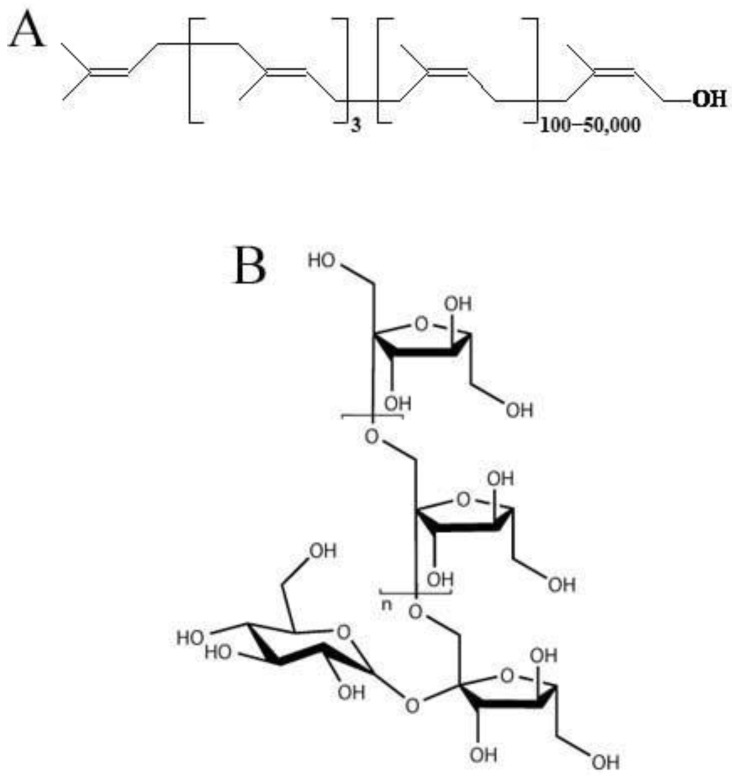
(**A**). Chemical structure of natural rubber. (**B**). Chemical structure of inulin.

Natural rubber (NR), a polymer composed of *cis*-1,4-polyisoprene (Figure 1A), is a very important raw material used in over 50,000 products [1] and is highly valued in industries such as transportation, medicine, and defense [2]. The amount and relative proportion of NR in rubber products has increased over the past 35 years. For example, in 1981, NR accounted for 30% of all rubber (natural and synthetic) used in the world; by 2013 this share had already increased to 42% [3]. This is because NR from plants is superior to petroleum rubber in several ways, e.g., the polymer of NR has a much higher molecular weight compared with synthetic rubber and the sustainable and renewable production of plant rubber is considered more efficient and environmentally friendly than the recycling of non-renewable petroleum [4,5].

Natural rubber has superior elasticity, tackiness, strength, thermal properties, elasticity, abrasion resistance, and impact resistance compared with synthetic rubber due to its unique molecular structure and high molecular weight (>1 million g/mol) [6]. Aircraft tires always contain a lot of NR. Truck tires are 30–40% NR and even car tires always contain a certain proportion (15–20%) of NR [6]. Natural rubber consists mainly of linear rubber polymers (*cis*-1,4-polyisoprene, Figure 1A) and secondary components (non-rubber components) that distinguish it from synthetic rubber. Non-rubber components include proteins and resins (fatty acids, triglycerides, sterols, etc.). These elements may also be involved in the construction of the rubber network, which may be the basis for the outstanding mechanical properties of NR [3].

According to numerous data from the literature, more than 2500 species of dicotyledonous plants can synthesize rubber [7]. The search for new rubber-bearing plant species remains relevant to this day. In our recent study in the flora of the Republic of Bashkortostan (Southern Ural region in Russia), we identified eight species of Asteraceae with a high content of natural rubber [8]. The role of rubber in protecting plants from herbivores and pathogens is generally recognized as the latex leaks out of the plant after injury. Many proteins produced in latex are clearly associated with defense, such as pathogenesis-associated polypeptides and chitinases. This evolutionary acquisition appears to have evolved independently several times, possibly to reduce herbivory and pathogen attacks [9]. However, no more than 10 plant species have been identified as a source of high-quality high-molecular-weight rubber that can be used on an industrial scale. The most famous of them are the Para rubber tree (*Hevea brasiliensis* (Willd. ex A.Juss.) Müll.Arg.), guayule (*Parthenium argentatum* Grey), krym-saghyz (*Taraxacum hybernum* Stev.), and the Russian (Kazakh) dandelion (*Taraxacum kok-saghyz* L.E. Rodin) (TKS) [5,10]. At the same time, only *H. brasiliensis*, cultivated mainly in the countries of the Asia-Pacific region, is the source of almost all NR in the world [11].

However, the global demand for NR is growing every year. Since NR yields are highly dependent on natural conditions, price volatility is added to climate change concerns. In the decade from 2001 to 2011, rubber prices tripled and then declined [11]. The global NR market in 2016 amounted to approximately USD 24 billion, with a volume of NR consumption of 12.9 million tons; by 2023 its forecast consumption will increase to 16.5 million tons (International Rubber Study Group (IRSG), http://natural-rubber.ru, accessed on 12 February 2023). Natural rubber demand could reach USD 68.5 billion by 2026 [12].

There is less and less suitable land for laying new *H. brasiliensis* plantations. In addition, these trees begin to produce rubber only after 7 years of cultivation. Furthermore, *H. brasiliensis* is susceptible to various infectious diseases, for example, caused by the well-known fungus *Microcyclus ulei* [11]. The small genetic diversity of *H. brasiliensis* grown on plantations also remains a difficult problem to solve [13]. It is unlikely that *H. brasiliensis* cultivation can be further expanded sufficiently to meet the growing global demand for NR due to both economic and environmental constraints [14]. It should also be borne in mind that, in order to meet the growing demand for rubber, it is necessary to destroy millions of hectares of tropical forests along with all their biodiversity. Moreover, *H. brasiliensis* is losing competition for land and labor against the economically more profitable African oil palm *Elaeis guineensis* [5]. Unfortunately, the supply of *H. brasiliensis* rubber is largely dependent on smallholders in Southeast Asia, so its industrial production is not subject to any global control and can quickly collapse under the influence of climate change and disease. Based on the understanding of these problems, the introduction of alternative rubber crops that provide geographic and genetic diversity on a global scale will positively affect the rubber trade while reducing price volatility and ensuring supply security [9]. By the beginning of the 21st century, many economically developed countries of the world realized the importance of finding alternative sources of NR. TKS, which has already been successfully grown on an industrial scale in the USSR and some other countries in the middle of the 20th century, takes first place in these searches. In the USSR, from one hectare of annual plantations of TKS, about 80 centners of roots were harvested; in terms of rubber, this is 176 kg per hectare [15]. TKS was first described in 1931 by the botanist of the Botanical Institute of the USSR Academy of Sciences L.E. Rodin after it was pointed out by local residents V. Spivachenko and V. Bukhanevich. Detailed information about the use of alternative sources of rubber in the USSR is given in [16]. In the 21st century, in many countries, the collection of germplasm resources began again and basic research on this plant began [17,18]. TKS belongs to the Asteraceae family and originates from the Tekes river basin near the mountainous border in the Tien Shan between Kazakhstan and China [19]. In Kazakhstan, the TKS range is associated with the intermountain valleys of the Kegen and Tekes rivers of the Eastern Tien Shan [20,21]. According to some reports, TKS also grows in Uzbekistan [2].

TKS grows most often in non-saline or slightly saline floodplain meadows and canals of agricultural land [22]. TKS can be cultivated in most temperate regions [23]. At the same time, it is possible to use fully mechanized farming in a standard annual crop [2], while *H. brasiliensis* plantations use manual labor. The most important thing is that the molecular characteristics and mechanical properties of TKS NR are similar to those of *H. brasiliensis* rubber. TKS rubber is of particular interest primarily to the tire industry due to its high molecular weight (polymer index) and fast crop maturation (six months for TKS versus seven years for *H. brasiliensis*) [3,5,17,24,25]. The relevance of alternative sources of NR can also be indicated by the fact that a number of companies have become interested and have already participated in or are participating in the implementation of projects with TKS; these are well-known companies such as American Sustainable Rubber, Bridgestone, Continental, Ford, Goodyear, KeyGene, Kultivat, Linglong, NovaBioRubber, and Sumitomo [2].

The roots of TKS contain about 2.89–27.89% NR (*cis*-1,4-polyisoprene) on a dry weight basis, with very similar macromolecular structure and composition characteristics to *H. brasiliensis* [22,26,27]. The rubber in TKS is also reported to have a higher molecular weight than *H. brasiliensis* [28].

In addition to high-quality rubber, the roots of TKS can produce a large amount of inulin (up to 40% of dry weight) [27,29], which is an important raw material for the food and medical industries, including bioethanol production. TKS roots also contain sucrose (10%), proteins (15%), cellulose (9%), hemicellulose (7%), lignin (5%), pectin (3%), and sterols (less than 1.7%) [3]. Inulin (Figure 1B) is the main storage carbohydrate in dandelions [30] and inulin carbon, if necessary, can be redirected towards rubber biosynthesis through acetyl-CoA, which occurs in nature towards the end of the growing season [31]. One study showed that in addition to rubber and inulin, TKS contains a wide variety of valuable metabolites, such as sesquiterpenoids, fatty acids and their derivatives, and phenolic compounds (caftaric and chicoric acids) [32]. It was pointed out that TKS leaves are a valuable source of chicoric acid, with potential use as an antioxidant for use as herbal medicine and nutrition for health food/fodder production [33].

However, TKS still has several inherent problems, such as the need for constant soil moisture during germination, slow growth rate, low competitiveness with weeds, significant rubber yield only at maturity, high heterozygosity, and self-incompatibility [2,5]. Therefore, the main efforts should be aimed at overcoming these shortcomings in TKS using speed breeding and biotechnology [14]. To develop strategies for breeding and genetic engineering of a given crop in order to improve its economically valuable traits, approaches are needed to accurately identify populations, lines, and varieties with different characteristics. All this is impossible without a thorough study of the genome and the genes involved in the regulation of the biosynthesis of rubber and other metabolites.

The earliest studies of the genetic diversity of TKS populations were carried out using the eSSR [24] and gSSR [5] methods. Due to the sequencing of the reference genome of TKS, Lin et al. [34] also made it possible to conduct genome-wide SNP studies and identify rubber biosynthesis genes. Since then, due to the continuous development of genomic research, more and more studies have been published on the analysis of TKS gene families at the genome level [35,36]. Moreover, according to many authors, TKS is the most suitable model plant for studying rubber biosynthesis, since it has a relatively small genome and a fast growth cycle [37]. Its genome is relatively small (1.29 GB) and contains 46,731 protein-coding genes [34]. In addition, TKS, unlike tree rubber-bearing plants, is relatively easy to genetically transform [38], so it can be used both for classical agrobacterium-mediated transformation and for CRISPR/Cas genome editing [39]. All this makes it possible to use TKS for genetic manipulations in order to study the molecular pathways that regulate the biosynthesis of NR, inulin, and other compounds.

Review articles on TKS genes involved in rubber biosynthesis have been previously published [2,6,40]. Since then, however, a number of new studies have been carried out. There is also a need to generalize all the data obtained, not only on the genes of rubber biosynthesis but also on other molecular genetic studies of TKS. Our review collected and analyzed data on advances in molecular genetics, genomics, transcriptomics, proteomics, and genetic engineering of TKS.

## 2. Molecular Genetic and Genome-Wide Studies

The simplest and cheapest methods for assessing the genetic diversity of plants include the methods of RAPD and ISSR analysis. For example, we used these methods to analyze the polymorphism of three TKS lines, two forms of *T. hybernum*, and some other dandelion species [18]. The RAPD primers OPD-07 and FS-10 are best suited for the detection of interspecific polymorphism, while the RAPD primers FS-10, OPC-05, and OPC-08 can be used for the detection of intraspecific dandelion polymorphism. The ISSR primers HB10, ISSR17, and ISSR36 were most effective for detecting interspecific polymorphism. For identification of the brown-achened and pinkish-achened forms of *T. hybernum*, primers HB10 and ISSR38 can be used. Primers HB10 and ISSR14 are best suited for identifying the three studied TKS lines.

More accurate data on plant genotypes can be obtained by analyzing microsatellite DNA polymorphisms. McAssey et al. [24] were the first to develop a large number of highly effective primers for the detection of SSR markers. In total, the authors were able to select primers for 767 loci, of which 192 pairs were tested in laboratory experiments. As a result, 48 primer pairs were effective for identifying single-locus polymorphisms. The best and most reliable 17 of these primer pairs were used to genotype 176 individuals from 17 natural TKS populations. The primers selected by these authors can also be successfully used for genotyping *T. officinale* (L.) Weber ex F.H.Wigg. [24] and *T. hybernum* [41]. In our further studies, it was shown that SSR primers TKS0091, TKS0097, TKS0107, and TKS0110 are suitable for identification of pinkish-achened and brown-achened forms of *T. hybernum*. Locus TKS0111 is suitable for unambiguous SSR identification of all three studied TKS lines [18].

Subsequently, studies were also carried out to search for new more universal SSR markers; for this, de novo sequencing was used [5]. The authors were able to select the 25 most convenient genomic SSRs (gSSR) and additionally, using previously known EST-SSR (eSSR) [24], the genotyping of TKS DNA samples from the germplasm bank and herbarium was carried out. It was shown that the TKS samples available in the collection were characterized by a rather low level of genetic diversity. It should be noted that 14 gSSRs in this study were also effective in the analysis of the genetic diversity of *T. officinale* samples and, according to the analysis of nucleotide sequences, they can also be used in the study of other Asteraceae species.

In the early days, genetic studies of TKS were complicated by the lack of information on the complete genome. The first draft TKS genome was sequenced and assembled by Lin et al. [34], using the PacBio SMRT sequencing method. The genome size was 1.29 Gb and 46,731 protein-coding genes were predicted. The large size of the genome was determined to a greater extent by repetitive DNA, for example, LTR-RT elements.

Since then, research on dandelion genomics has continued to a new level. For example, sequencing of 58 TKS samples was carried out, which, according to the results of the analysis, were divided into three large groups: Zhaosu County in Xinjiang (populations A and B); Tekes county in Xinjiang (population C); Lake Tuzkol in Kazakhstan (population D). The D population showed a closer genetic relationship with the C population, compared with the AB populations. A comparative analysis of the genomes of different populations showed differences in the genes involved in their adaptation to different environmental conditions [42]. In another study, high-quality genomes of the rubber-bearing TKS and the medicinal plant *T. mongolicum* Hand.-Mazz. were sequenced and assembled. For this, PacBio SMRT sequencing technologies, BioNano optical-mapping, and Hi-C technologies, supplemented with the Illumina whole-genome shotgun data, were used [43]. The genome of TKS was expected to be larger and to contain more genes associated with rubber biosynthesis. The largest TKS-specific segment was presence–absence variations (PAV), with a size of 101,040 kb on chromosome 1, which contained 18 genes, including three genes encoding puromycin-sensitive aminopeptidases necessary for maintaining cell growth and viability, as well as two genes encoding NB-ARC domain-containing disease-resistance proteins. At the same time, a small specific fragment of the *T. mongolicum* genome did not contain any genes. To compare genes in the pathway associated with rubber biosynthesis, a total of 90 and 76 candidate rubber biosynthesis genes encoding 21 enzymes were identified in the genomes of TKS and *T. mongolicum*, respectively. TKS-specific rubber biosynthesis genes encoding *cis*-prenyltransferase (CPT), 4-hydroxy-3-methylbut-2-enyl diphosphate reductase, A-cholesterol acyltransferase, and mevalonate kinase (MVK) have been discovered. A comparison between NR initiation and elongation genes showed that TKS has a greater number of latex-expressed genes, whose products are involved in both the initiation and the elongation of NR chains.

For TKS, there are still no widely available SNP platforms for genome-wide association studies’ (GWAS) analyses and searches for high-yielding lines, populations, and individuals. However, research in this direction is already underway. For example, in 2017, 21,036 SNPs were identified in the TKS root transcriptome. Of these, 50 SNPs in 39 transcripts were associated with rubber biosynthesis [17]. More rigorous analyses of trait variance, correlation between traits, and heritability of traits associated with rubber yield were also conducted for association with 42 SNP markers found on genes for rubber biosynthetic enzymes [14]. SNP263, SNP375, SNP912, SNP1006, and SNP1245 were significantly associated with rubber concentration, with a *p*-value of less than 0.05. Of these, four markers were located in the genes of enzymes of the 2-C-methyl-D-erythritol-4phosphate pathway (MEP).

In 2021, a total of 524,812 SNPs were found, based on analysis of whole genome sequencing results [42]. These SNPs were used to construct the population structure, genetic diversity, and evolutionary analysis of 58 wild TKS plants collected from four different regions.

Recently, Yang et al. [44] created a special TKS population by crossing lines with low and high rubber content and, based on the results of whole genome sequencing of this hybrid population, created a genetic map, including 322,439 SNPs and a number of rubber content QTLs. Such genetic maps are planned to be used in the creation of modern test systems for the marker-assisted selection of TKS. The results of all these studies have laid the foundation for future large-scale QTL mapping and GWAS that will be used in the genomic selection of TKS for high agronomic performance and rubber yield.

A large problem remains concerning the selection of accurate molecular markers that would make it possible to distinguish valuable species of rubber-bearing dandelions from weeds. One solution can be the analysis of DNA markers in the chloroplast genome. Moreover, knowledge of the nucleotide sequences of the chloroplast genome makes it possible to restore phylogenetic relationships between different maternal dandelion species. Based on these considerations, Zhang et al. [45] sequenced the chloroplast genomes of TKS (151,338 bp), *T. officinale* (151,299 bp), and *T. brevicorniculatum* Korol. (TB) (151,282 bp). As expected, TKS was closer to TB than to *T. officinale*. Of the other Asteraceae species, *Lactuca sativa* L. was the closest to dandelions. The authors rightly believe that the results of their study can be used to differentiate and purify germplasm from weedy species, as well as to study the potential gene flow among Taraxacum species. To do this, it is proposed to use a number of SNP markers found both in the chloroplast and in the nuclear genome that distinguish these three dandelion species.

## 3. Rubber Biosynthesis Genes

The most important aspect that TKS breeders should consider is obtaining genotypes with more intensive and stable rubber accumulation, which will help make this plant a more profitable rubber-bearing crop. Determining both classical and biotechnological breeding strategies requires a better understanding of rubber biosynthesis and its control in TKS, as well as the identification of rate-limiting enzymes and competing metabolic pathways for rubber biosynthesis [46].

An early study showed that the small rubber particle protein TkSRPP3 plays an important role in rubber biosynthesis [47]. Transgenic TKS plants with an increased level of expression of the *TkSRPP3* gene were characterized by an increased accumulation of rubber. At the same time, the TKS lines with an induced RNA interference of the *TkSRPP3* gene were distinguished not only by a significantly reduced content of rubber but also by a decrease in its molecular weight.

**Figure 2:**
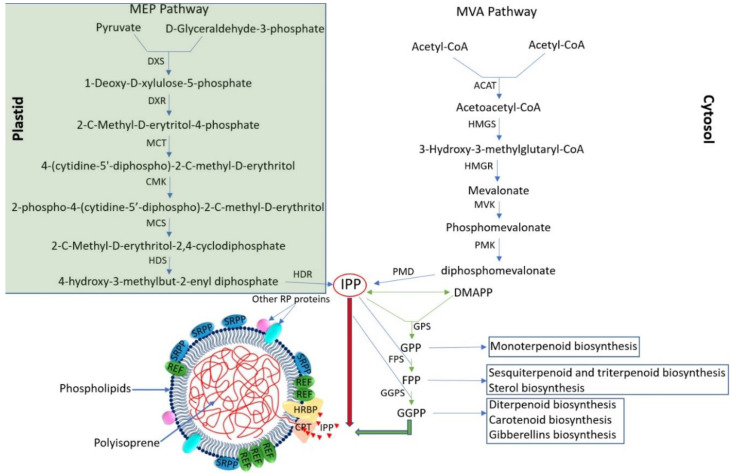
The pathway of natural rubber, 2-C-methyl-D-erythritol-4phosphate (MEP) and mevalonate (MVA) in *Taraxacum kok-saghyz*. DXS—1-Deoxy-D-xylulose-5-phosphate synthase; DXR—1-Deoxy-D-xylulose-5-phosphate reductoisomerase; MCT—2-C-Methyl-D-erytritol-4-phosphate cytidyl transferase; CMK—4-(cytidine-5′-diphospho)-2-C-methyl-D-erythritol kinase; MCS—2-C-methyl-D-erythritol-2,4-cyclodiphosphate synthase; HDS—4-hydroxy-3-methylbut-2-enyl diphosphate synthase; HDR—4-hydroxy-3-methylbut-2-enyl diphosphate reductase; ACAT—acetyl-coenzyme A acetyltransferase; HMGS—3-hydroxy-3-methylglutaryl coenzyme A synthase; HMGR—hydroxymethylglutaryl coenzyme A reductase; MVK—mevalonate kinase; PMK—phosphomevalonate kinase; PMD—diphosphomevalonate decarboxylase; IPP—isopentenyl pyrophosphate; DMAPP—dimethylallyl pyrophosphate; GPS—geranyl pyrophosphate synthase; GPP—geranyl pyrophosphate; FPS—farnesyl pyrophosphate synthase; FPP—farnesyl pyrophosphate; GGPS—geranylgeranyl pyrophosphate synthase; GGPP—geranylgeranyl pyrophosphate; CPT—*cis*-prenyltransferase; HRBP—HRT1-REF bridging protein; REF—rubber elongation factor; SRPP—small rubber particle protein; RP—rubber particle. According to [6,60].

Natural rubber is composed of isopentenyl monomers derived from isopentenyl pyrophosphate (IPP) synthesized primarily by the cytosolic mevalonic acid (MVA) pathway as well as the plastid MEP pathway (Figure 2) [6]. New rubber molecules are initiated by the binding of an allyl pyrophosphate, usually cytoplasmic farnesyl pyrophosphate (FPP), and then the rubber is polymerized from a hydrophilic monomeric substrate, IPP, which is produced by both pathways (MVA and MEP) [9]. More than five thousand IPPs make up the chains of high-molecular-weight polymers of NR (*cis*-1,4-polyisoprene, >106 kDa) [48]. To date, a total of 90 functional genes have been identified that control the rubber synthesis pathway in TKS, including 32 genes in the MVA pathway, 19 genes in the MEP pathway, 17 genes in rubber synthesis initiation, and 22 genes in rubber chain extension. In addition, many transcription factors have been identified that regulate rubber biosynthesis; however, so far they have not been described in TKS but have been described in other rubber-bearing plants [44]. Isopentenyl pyrophosphate is primarily produced by MVA and is the main building block of not only rubber, but many other secondary metabolites such as monoterpenoids, sesquiterpenoids, triterpenoids, and steroids, which play an important role in plant growth, development, and defense against phytopathogens and herbivores [31]. The MVA pathway generates IPP through six major catalytic steps, using the following enzymes: acetyl-coenzyme A acetyltransferase (ACAT), 3-hydroxy-3-methyl-glutaryl coenzyme A synthase (HMGS), hydroxymethylglutaryl coenzyme A reductase (HMGR), MVK, phosphomevalonate kinase (PMK), and mevalonate diphosphate decarboxylase (MVD). Among these enzymes, HMGR is the key rate-limiting enzyme of the MVA pathway (Figure 2) [49,50,51]. Rubber forms on the surface of rubber particles dispersed in the latex of TKS roots [37]. Rubber *cis*-prenyltransferases (CPTs), which catalyze rubber chain extensions, are the main component of the rubber transferase (RTase) complex on the surface of rubber particles [52]. IPP, and its isomer dimethylallyl pyrophosphate (DMAPP), can be catalyzed by short chain prenyltransferases to form prenyl diphosphate intermediates, including geranyl pyrophosphate (GPP), FPP, and geranylgeranyl pyrophosphate (GGPP), which can initiate in vitro rubber biosynthesis. In the biosynthesis of high-molecular-weight rubber, in addition to CPTs, *cis*-prenyltransferase-like proteins (CPTLs), rubber elongation factors (REFs), and SRPPs are also involved (Figure 2) [53]. In TB, a homologue of the human *NgBR* gene, the *TbRTA* gene, was also found, the product of which is an important component of the RTase complex, since its knockdown strongly disrupts rubber biosynthesis [54]. More detailed information on all known genes and proteins involved in rubber biosynthesis can be obtained from a review article by Cherian et al. [2].

The assembly of the first TKS genome revealed the presence of eight *TkCPT* genes and two *TkCPTL* genes [34]. Phylogenetic analysis showed that all four *CPT* genes of type I in the assembled TKS genome were located in the group 1, which also contains genes for three *T. brevicorniculatum CPTs*, two *L. sativa CPTs*, and two *H. brasiliensis CPTs*, suggesting the importance of this group for rubber biosynthesis. It has been shown that the *TkCPT1* and *TkCPT2* genes are predominantly expressed in latex. One *TkREF* gene and nine *TkSRPP* genes have also been found in the TKS genome.

The next genome-wide analysis also showed that TKS has nine SRPP genes (*TkSRPP1-9*) and one REF gene (*TkREF1*) [55]. All proteins of the TkSRPP family were predicted to have a cytoplasmic location, while the TkREF1 protein was predicted to have a transmembrane domain. Based on the results of phylogenetic analysis of the *SRPP/REF* genes of TKS and other plants, five subgroups were identified. Proteins of the TkSRPP/REF family were distributed into four of five subgroups, with most of them belonging to subgroups III and IV, as well as in *L. sativa*, while in *H. brasiliensis* only a few proteins of this family belonged to subgroups III and IV. The promoters of the *TkSRPP/REF* genes have been predicted to contain many *cis*-elements sensitive to hormones and abiotic stress. The authors also experimentally proved that treatment with methyl jasmonate (MeJA) increases the expression levels of the *TkSRPP3-4* and *TkREF1* genes. It was previously reported that the *TkSRPP2* gene was cloned, its localization in the cytoplasm was shown, and its high expression was found in 6-month-old TKS roots [56].

The ontogenesis of rubber particles most likely proceeds similarly to secretory protein pathways, including the rough endoplasmic reticulum and the Golgi apparatus. Indeed, using *H. brasiliensis* as an example, it has been shown that REFs, SRPPs, soluble CPTs, and bridging proteins (HRBP or CBP) are associated with the endoplasmic reticulum. The biosynthesis of NR occurs in the latex (the cytoplasm of highly specialized cells, laticifers) [57]. The vacuoles appear to be the storage for mature rubber particles, but rubber biosynthesis and true particle growth take place in the cytosol. The biosynthesis of rubber is catalyzed by the rubber polymerization complex embedded in the rubber particle membrane and cannot take place inside the vacuole [9].

Another way to elucidate the biological functions of rubber biosynthesis genes can be to study the tissue-specific and inducible expression of their promoters. For this purpose, the promoter sequence of the geranylgeranyl pyrophosphate synthase (GGPPS) gene was cloned from TKS in the pCAMBIA1304 binary vector with the *GFP* reporter gene. The functionality of the resulting vector was proven on cells of the onion epidermis. It was planned to use this construct to study tissue-specific and inducible expression of the *GGPPS* gene and hence the biosynthesis of rubber [58].

For an analysis of the *TkSRPP* gene promoter, it was fused in various deletion variants with the *GUS* reporter gene [59]. Then, it was shown on transgenic tobacco plants that the full-length promoter is not tissue specific, but that the core part of the promoter induced reporter gene expression under the action of MeJA, abscisic acid (ABA), and salicylic acid (SA), as well as wounds.

## 4. Genes for Inulin Biosynthesis and Other Genes

**Figure 3:**
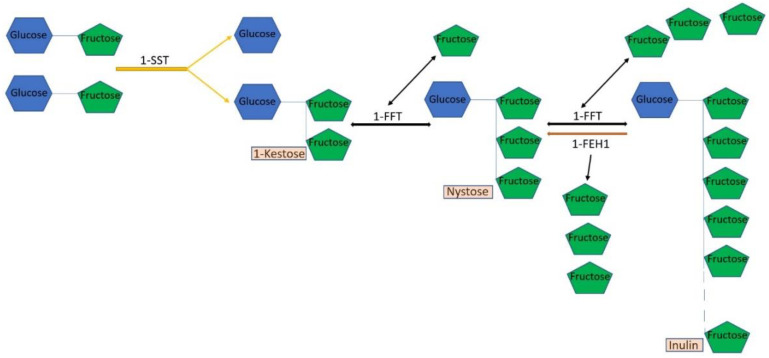
Biosynthesis of inulin in *Taraxacum kok-saghyz*. 1-SST—sucrose:sucrose-1-fructosyltransferase; 1-FFT—fructan:fructan-1-fructosyltransferase; 1-FEH1—fructan-1-exohydrolase. Taken from [61].

In addition to rubber, inulin (Figure 1B) is also a valuable metabolite of TKS. Therefore, it is not surprising that the genes of the biosynthesis of this polymer in TKS are also already well known. Inulin synthesis in plants is regulated by several fructosyltransferases, of which the most important are sucrose:sucrose-1-fructosyltransferase (1-SST) and fructan:fructan-1-fructosyltransferase (1-FFT) (Figure 3). Inulin cleavage is catalyzed by fructan-1-exohydrolase (1-FEH1), which sequentially hydrolyzes terminal fructose residues from fructan molecules (Figure 3). The highest level of expression of the three main inulin biosynthesis genes (*1-SST*, *1-FFT,* and *1-FEH1)* is found in the roots of TKS, compared with other organs. The addition of low concentrations of naphthylacetic acid to the nutrient medium stimulates inulin biosynthesis in suspension cultures of TKS due to a positive effect on the expression level of the *1-SST* and *1-FFT* genes [61]. It is known that, in dandelions, excess free sugars (fructose and sucrose), which are formed during the degradation of inulin, can be used to synthesize IPP, which increases the production of polyisoprene. Even Soviet researchers have shown that wild-growing TKS is able to accumulate rubber in greater quantities when the level of inulin decreases [62]. Similar data, on the other hand, were shown in studies by Post et al. [52] using the example of TB, which accumulated more inulin due to inhibition of the activity of the enzyme CPT (which catalyzes polyisoprene chain elongation), which ultimately led to a decrease in rubber content. It was also shown that overexpression of the *1-FEH1* gene in transgenic TKS and TB led to a twofold increase in the rubber content in the roots of these plants [63]. CRISPR/Cas9 editing of the TKS genome, during which the *1-FFT* gene is knocked out, may also lead to an increase in rubber content [39]. Obviously, the pathways of rubber and inulin biosynthesis in dandelion roots are in antagonistic relationships, primarily due to competition for the same photoassimilates. Moreover, the degradation of inulin is considered the main source of the formation of new acetyl-CoA, which, in turn, is used to produce MVA and polyisoprene [63]. That is why many studies of inulin biosynthesis genes are carried out in the context of their influence on rubber biosynthesis. For example, a comparative analysis of the transcriptomes of TKS lines with increased (HR) and decreased (LR) rubber accumulation showed that there was no difference between these lines in the level of expression of the *1-FEH1* gene. However, in HR lines, an increase in the content of transcripts of the gene encoding sucrose-1-fructosyltransferase (*1-SST*) was found, while the expression level of the fructan-1-fructosyltransferase (*1-FFT*) gene in these plants was suppressed [31].

After the full genome annotation of TKS, genome-wide studies of entire gene families were begun, which are very useful for future work on the genetic engineering of this plant. For example, in *T. kok-saghyz*, 22 *TkSWEET* genes coding for sugar transporter proteins were identified by a search for homologues using bioinformatic approaches. Using RNA-seq and qRT-PCR methods, it was shown that all these genes have overlapping yet still different expression patterns in different plant tissues. In the latex of rubber-bearing dandelions, the *TkSWEET1* and *TkSWEET12* genes were primarily expressed and their protein products were localized on the plasma membrane [64]. On yeast mutants, when studying heterologous expression, it was proven that only TkSWEET1 exhibited sugar transporter activity, with a preference for monosaccharides.

Studies on the transcription factor *WRKY* genes were also started and, in TKS, 72 *WRKY* genes were identified. Exon–intron structures of these genes were compiled, which allowed them to be divided into three main groups. Moreover, transcriptome analysis showed that all these genes responded differently to abiotic stress factors. For example, the *TkWRKY18*, *TkWRKY23*, and *TkWRKY38* genes were significantly upregulated during cold stress, while the *TkWRKY21* gene was upregulated under heat stress [65].

TKS is naturally well-suited for growing in the temperate climates of Central Asia, China, and southern Russia. However, when moving this plant to the north, problems arise associated with insufficient cold tolerance. For example, even in the agriculturally developed Volga–Ural region of Russia, an increase in the cold tolerance of TKS is required, since frosts are quite possible here in late May and late August. According to our observations, the planted TKS in the conditions of the Republic of Bashkortostan never survives in winter, which indicates a rather low winter hardiness of this plant compared with other dandelion species. Cold acclimatization can speed up the germination period of TKS in spring and delay the wilting period in autumn, which will increase rubber production by extending the growing season. Transcription factors CBF, which are encoded by a whole family of highly conserved genes, play a key role in plant adaptation to cold stress. Using bioinformatic approaches, 10 *CBF* genes (*TkCBF1-TkCBF10*) were found in the TKS genome, their phylogenetic relationships and protein conservative motifs were determined, and the physicochemical properties of proteins, the structure of genes, and the composition of promoter *cis*-regulatory elements were predicted [66]. These authors also determined the expression profile of the *TkCBF1-TkCBF10* genes after cold treatment. An increase in the expression level was found for different times of cold treatment in the *TkCBF2*, *TkCBF5*, *TkCBF7*, *TkCBF8*, and *TkCBF10* genes. The obtained results can be used in planning works on obtaining transgenic and CRISPR-edited TKS plants with increased cold tolerance.

Protein encoding by histone-modifying genes (*HMGs*) catalyze a number of post-translational modifications that affect chromatin organization and conformation, which in turn regulate many downstream processes, including gene expression. Panara et al. [1] identified 154 putative homologues of *HMGs* genes in TKS: 60 histone methyltransferases (*HMT*), 34 histone demethylases (*HDM*), 42 histone acetyltransferases (*HAT*), and 18 histone deacetylases (*HDAC*), with TKS surpassing other plants in the number of *HAT* and *HMT* genes (for example, *Medicago truncatula* and *Arabidopsis thaliana*). Additionally, expression patterns in various tissues were determined for a number of *HMGs* genes. For example, in latex, an increased level of expression of the *TksSDG28* gene encoding one of the TKS *HMTs* was found and the *TksHAG10* gene was abundantly expressed in TKS roots. This study describes for the first time the genomic organization of the *HMGs* genes and the data obtained can become a prerequisite for the functional characterization of epigenetic modifications of genes involved in rubber biosynthesis.

Based on the fact that rubber biosynthesis is positively regulated by MeJA, an analysis of DEGs (differentially expressed genes) in TKS roots under the action of this phytohormone was carried out. A total of 7452 DEGs compared with the control were identified, with seven transcription factor genes associated with natural rubber biosynthesis in latex tissue identified [67]. Of these, the *TkDREB2* gene was also activated by drought and could stimulate rubber biosynthesis through its effect on *TkHMGR*. In an earlier study, the jasmonate-inducible *TkDREB2* gene was cloned and used to create transgenic tobacco plants that showed high drought tolerance and antioxidant system activity [68].

In TKS laticifers, in addition to rubber, various terpenes are also accumulated and, in order to improve the production of rubber, it is of interest to reduce the amount of these terpenes. For this, van Deenen et al. [69] created TKS plants with suppressed expression of oxidosqualene cyclase (TkOSC1) due to RNA interference, which led to a decrease in the content of terpenes in rubber by 67%. The TkOSC1 enzyme was identified by the authors in their previous study [70], where its involvement in triterpenoid biosynthesis was reported.

In order to search for candidate genes involved in the regulation of TKS root size, a comparative transcriptomic analysis was performed, with different sizes and shapes of roots [71]. Several DEGs were further tested by qRT-PCR and expression of the *TkBG11* encoding β-1,3-glucanase 11 and the *TkTIFY10A/JAZ1* encoding jasmonate ZIM-domain protein 1 was found to correlate with the corresponding root morphotype.

One of the obstacles to the introduction of TKS into industrial production is its self-incompatibility, so the search for genes that regulate pollination processes has begun. Wollenweber et al. [72], by studying DEGs in cross-pollinating and self-pollinating plants, identified three candidate pollination regulator genes: *LRX, TUBB,* and *XTH33*. The *LRX* family is involved in maintaining the integrity and assembly of pollen tube cell walls. *TUBB* encodes β-tubulin and *XTH33* encodes xyloglucanendotransglycosylase, which is involved in cell wall biogenesis.

In TKS, seven ascorbate peroxidase genes (*TkAPXs*) were identified, the expression level of most of which in roots and leaves was higher than in flowers and latex [73]. The expression of these genes was induced under the action of abiotic stress and the action of the ethylene and MeJA.

TKS mutants for the *TkRALFL1-Like1* gene were obtained by the CRISPR/Cas method, which differed in a tap root phenotype that is easier to grow and harvest, as well as higher root biomass and higher yields of inulin and natural rubber [74].

## 5. TKS Transcriptomic and Proteomic Studies

Hundreds of genes are simultaneously involved in all biological processes in plants, so the most effective way to identify them and to simultaneously evaluate their functions is the sequencing and analysis of transcriptomes. Comparative transcriptomic studies of TKS lines with different accumulations of NR will help elucidate which genes are involved in the synthesis, regulation, and accumulation of this natural polymer and can help turn TKS into an economically viable rubber crop through genetic engineering manipulations.

One of the first TKS root transcriptomes was sequenced by the Illumina method and 55,532 transcripts over 200 bp were obtained and 21,036 SNPs were identified [17]. Of these, 50 SNPs were found in 39 transcripts with high homology to 49 genes associated with rubber biosynthesis. The DNA markers discovered during this study became the basis for further work on the genotyping of this plant.

A 454 pyrosequencing method was also used to sequence the TKS root transcriptome [46]. In this study 73,350 UniGenes were identified, of which 50,383 were annotated. Subsequent differential gene expression analysis between HR and LR lines revealed a large number of genes, including those involved in rubber biosynthesis.

A recent study by Yang et al. [31] aimed to identify DEGs in HR and LR rubber TKS lines. The authors used the nanopore sequencing method in their study, which made it possible to identify a much larger number of expressed genes in TKS roots than in previous studies. As a result, 3134 DEGs were identified. Among these genes, 1157 DEGs were upregulated and 1977 DEGs were downregulated in HR lines compared with LR lines. The HR lines showed mainly genes involved in rubber biosynthesis, while the LR lines had activated genes involved in the synthesis of squalene, phenylpropanoids, fatty acids, and ketones, which require the same substrates (IPP or acetyl-CoA) as biosynthesis of rubber. As for the rubber biosynthesis genes, the lines with a high content of rubber showed an increased content of transcripts of the *CPT1* and *CPTL1* genes, while the activities of the *CPT2*, *3*, *6*, *7*, *8,* and *CPTL2* genes were low in both TKS lines [31]. Indeed, the knockdown of *TkCPTL1*, which is predominantly expressed in latex, led to the abolition of rubber synthesis. On the contrary, the suppression of *TkCPTL2* showed no change in the TKS phenotype, which confirms the importance of the *TkCPTL1* gene for rubber biosynthesis [75]. The highest content was shown for *SRPP* gene transcripts, of which eight genes were identified in total. Lines with a high content of rubber were characterized by a high level of expression of the *SRPP4*, *5*, *6*, and *9* genes. The HR lines also showed a higher expression level of the *REF1* gene compared with the LR line [31]. These results make it possible to plan further work on the activation and suppression of the expression of certain genes by classical genetic engineering and genome editing in order to increase rubber production. Additionally, based on the obtained results, it is possible to plan the selection of high-yielding TKS populations and lines based on the results of real-time RT-PCR of various genes for the biosynthesis of rubber and other metabolites.

Of great interest is the study of not only root, but also leaf metabolites of TKS. Some information in this area can be obtained by studying the transcriptome. For example, the TKS transcriptome has been studied after exogenous treatment with ethephon (a precursor of ethylene), which has hormonal activity and can influence both primary and secondary metabolism. As a result of the studies, an increase in the number of ribosomes, an increase in the expression in the genes of glycolysis/gluconeogenesis, the biosynthesis of phenylpropanoids, and the metabolism of linoleic acid, lysine, starch, and sucrose were revealed [76].

Not all transcribed genes are eventually translated into proteins, so further studies of the mechanisms of the regulation of rubber biosynthesis should also focus on proteome analysis. In their first study, Xie et al. [13] identified 371 proteins in mature TKS roots using 2-DE gel electrophoresis and mass spectrometry. A total of 3545 individual proteins were detected in growing roots using high-resolution mass spectrometry (large-scale shotgun analysis, Shotgun MS). It has been predicted that many of these proteins are involved in the process of carbon metabolism with catalytic activity in membrane-bound organelles using the KEGG (Kyoto Encyclopedia of Genes and Genomes) and GO (the Gene Ontology resource) databases. Many proteins have also been identified with the ability to bind, transport, and biosynthesize phenylpropanoids. This study also identified 58 proteins involved in NR biosynthesis, including eight SRPPs and REFs involved in both pathways of rubber biosynthesis: MVA and MEP.

Comprehensive studies of the TKS transcriptome and proteome may be promising. Such a study, by Xie et al. [48], identified 34,710 genes and 3695 proteins. Among them, 22,194 genes and 2145 proteins were differentially expressed during root development. In the course of this study, the authors were able to identify 102 genes and proteins associated with the biosynthesis of NR, for example, 10 CPTs, 8 SRPPs, 2 REFs, and 1 HRT1-REF protein (HRBP). Analysis of DEGs and differentially abundant proteins (DAPs) showed that the protein products of the *CPT7*, *SRPP5*, *SRPP6*, and *SRPP9* genes most likely play the largest role in the regulation of rubber biosynthesis [48].

To study the biological role of latex due to the expression of barnase under the control of the REF promoter, latex non-exuding TKS were obtained and a comparative analysis of the proteome and metabolome of these plants with the wild type was carried out [77]. In this study, 8106 proteins were identified and many proteins involved in the biosynthesis of rubber, inulin, and other TKS metabolites were described in detail. In another study, combined with Pro-Q Glycoprotein gel staining and mass spectrometry techniques, profiling of the rubber latex glycoproteomics was presented and, finally, 144 species of glycosylated proteins were identified, including 65 DAPs after ethylene treatment. In the course of the study, one of the HMGS2 isoforms was found, which was abundantly expressed in latex in response to ethylene, and overexpression of the *HbHMGS2* gene in TKS contributed to an increase in rubber accumulation [78].

## 6. Genetic Transformation of TKS

Since TKS is both a model object in the study of rubber biosynthesis and potentially valuable for agro-industrial production, it is of great interest to develop methods for the genetic transformation of this plant. One of the publications in this field was the study of Zhang et al. [38], who reported on the successful testing of a method for transforming TKS root explants using *A. rhizogenes* strain *K599* carrying a binary vector with genes for kanamycin resistance and green fluorescent protein. A hormone-free nutrient medium was used when transgenic plants of TKS and its relative TB were obtained. The average transformation efficiency (number of transgenic plants/number of root explants) was 24.7% and 15.7% for TKS and TB, respectively.

A number of studies were devoted to testing RNA interference technologies in transgenic TKS plants to research the functions of rubber biosynthesis genes. Changes in the expression levels of *SRPP* genes in transgenic TKS induced by RNAi technology demonstrated qualitative and quantitative changes in the course of rubber biosynthesis in these plants [47]. RNAi technology was also used to study the role of *cis*-prenyltransferases by changing the expression levels of three different genes from the prenyltransferase family in the transgenic TB [52]. The silencing of polyphenol oxidase genes in transgenic TKS and *T. officinale* was previously studied using the same RNA interference technology [79].

In recent years, the CRISPR/Cas genome editing method has been increasingly used to block the expression of the studied genes instead of using RNAi technology. As mentioned above, the TKS genome was edited by delivering CRISPR/Cas components using *A. rhizogenes* as part of a binary vector for the knockout of the *FFT1* gene [39]. Another study was devoted to the knockout of the *TkRALFL1-like1* gene by the CRISPR/Cas method [74].

**Figure 4:**
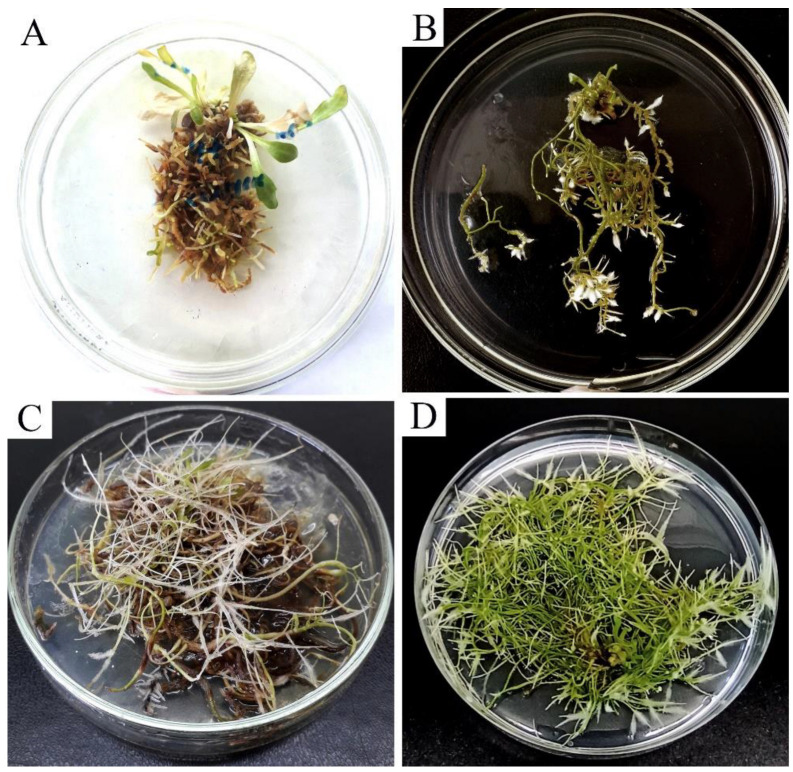
TKS hairy root cultures (our studies). Spontaneous regeneration of shoots was often observed in hairy roots (**A**,**D**). Green hairy roots often developed (**B**,**D**). With long cultivation, the roots of some lines turned brown and died (**C**).

In order to study the possibility of the biotechnological production of NR, we have developed methods for inducing hairy roots not only in TKS but also in *T. hybernum* [80,81]. In these studies, we used different types of explants, which were infected with *A. rhizogenes* strains A4 and 15834 under various inoculation conditions. The highest efficiency of transformation by both strains was observed when pricked with a needle containing a suspension of agrobacteria into the hypocotyls. It was shown that the A4 strain was more effective in the transformation of TKS than 15834 [80]. Shoot regeneration was constantly observed from hairy roots (Figure 4), which proved the possibility of creating transgenic TKS using *A. rhizogenes*. These studies were continued and hairy roots were obtained, accumulating an average of 7.5% rubber per dry weight, which is comparable to the roots of TKS grown in the field [82]. These data may indicate the possibility of using TKS hairy roots for rubber production in the future. However, to date, this technology seems to be economically unprofitable.

As for the transformation of *A. tumefaciens*, only a few works in this field are known for TKS. For example, transgenic TKS plants were created by transforming leaf discs with strain EHA105, where 6-benzylaminopurine and indoleacetic acid were used for plant regeneration [47]. CRISPR/Cas TKS mutants for the *TkRALFL1-like1* gene were also created using *A. tumefaciens* [74]. Genetic transformation of dandelions appeared to be more efficient with *A. rhizogenes* than with *A. tumefaciens* [83]. However, it should be borne in mind that, during the transformation of *A. rhizogenes* into plants, in addition to the target genes, *rol*-genes are also transferred, and in the future, it will become necessary to select plants without these genes. Therefore, in the laboratory of K. Cornish, studies were carried out to identify the phenotypic manifestations of *rol*-genes in TKS in order to further successfully select plants according to their phenotype that do not contain or do not express these transgenes [83].

TKS is also used as a model object for the analysis of promoters of rubber biosynthesis genes from *H. brasiliensis*. For example, in transgenic TKS plants, the activity of the *HbPEP16* gene promoter was studied and it was found that it was induced in laticifers in response to ABA, MeJA, 1-Naphthaleneacetic acid, salt, light, and darkness. The authors proposed to use the *HbPEP16* promoter to regulate the expression of rubber biosynthesis proteins in laticifers [84].

Recently, a technology for the genetic transformation of TKS without in vitro cultivation was proposed, which was called the cut–dip–budding delivery system [85]. To do this, plants whose roots have been partially cut out are placed for a short time in a suspension of *A. rhizogenes*, and then transgenic shoots are regenerated from the obtained genetically transformed hairy roots on nutrient-rich non-sterile soil. Thus, no in vitro culture is used at any of the stages, which greatly facilitates the genetic transformation of TKS.

## 7. Conclusions

The modern production of NR from *H. brasiliensis* is threatened by a large number of negative factors such as pathogenic microorganisms, strict climatic requirements for plantations, the rapid growth of world demand, the replacement of rubber plantations with African oil palm trees, and the destruction of natural forest ecosystems [3]. TKS is considered a promising alternative source of rubber for growing in the temperate zone. High heterozygosity, poor vigor, low competitiveness in the field, and inbreeding depression remains an obstacle to the widespread commercial cultivation of TKS [44]. Despite many years of breeding, TKS has not been completely domesticated and is characterized by low yields [86]; thus, in the 21st century, research has begun on the genetic engineering improvement of this plant. Although the TKS genome has been sequenced and annotated and most of the genes involved in rubber biosynthesis have been identified, only some research papers have been published to date reporting the successful introduction of mutations in the *1-FFT* and *TkRALFL1-Like1* genes by the CRISPR/Cas method. Due to the development of a new prime editing method, it has become possible to make the necessary changes directly in the genes of rubber biosynthesis; the results of such studies have not yet been published. In addition, one of the approaches to increase productivity and competitiveness with weeds in TKS could be its hybridization with other species of dandelions; such work is already underway [87]. Another way to increase the content of rubber in the roots of TKS can be its autopolyploidization using colchicine, which was conducted repeatedly in the middle of the 20th century. In the 1940s, tetraploid forms of TKS were obtained at least twice [88,89,90]; however, polyploids were characterized mainly by an increased size of roots and seeds, while rubber content high values were not recorded. Recently, Luo et al. [86] repeated this work and, in tetraploid plants, an increase in the concentration of rubber by 47.7% and a decrease in the concentration of inulin/sugars were observed. In TKS selection, chemically induced mutagenesis can also be used. We previously obtained mutant TB lines using sodium azide, which accumulated twice as much rubber in their roots as the wild type [91].

The lack of an environmentally friendly and cost-effective industrial-scale proven rubber extraction process remains another significant problem for the development of TKS into an industrial crop [92]. For rubber extraction, we mainly use two methods in the laboratory: extraction with organic solvents [3] or a microbiological approach [93]. However, for industrial production, it is necessary to develop cheaper methods to maintain the high quality of TKS rubber, which is the subject of a detailed review [92].

Thus, further studies of the biosynthesis of NR under both in planta and in vitro conditions in various biological systems, such as bacteria, yeasts, and plant cells, as well as in cell-free systems, are needed [12], involving new species of rubber-bearing plants. In the long term, rubber from TKS could complement the market share currently held by various synthetic rubbers, with a significant reduction in carbon footprint.

## Data Availability

The data presented in this study are openly available at https://doi.org/10.1134/S0033994619030105, https://doi.org/10.1080/23818107.2022.2147998, https://doi.org/10.1007/s10722-021-01233-1, https://doi.org/10.18699/VJ18.337, https://doi.org/10.3117/plantroot.11.64, https://doi.org/10.3329/ptcb.v27i2.35019, https://doi.org/10.31301/2221-6197.bmcs.2020-36, https://doi.org/10.31301/2221-6197.bmcs.2020-11.
